# The Impact of Time-Restricted Meal Intake on Glycemic Control and Weight Management in Type 2 Diabetes Mellitus Patients: An 18-Month Longitudinal Study

**DOI:** 10.7759/cureus.53680

**Published:** 2024-02-06

**Authors:** Smriti Rastogi, Narsingh Verma, Gourav S Raghuwanshi, Virendra Atam, Dileep Kumar Verma

**Affiliations:** 1 Physiology, King George's Medical University, Lucknow, IND; 2 Physiology, People's College of Medical Sciences and Research Centre, Bhopal, IND; 3 Internal Medicine, King George's Medical University, Lucknow, IND

**Keywords:** time-restricted meal intake, biochemical parameters, anthropometric, fasting and post prandial blood sugar, chronomedicine, fasting, chrononutrition, chronobiology, endocrinology and diabetes, diabetes mellitus type 2

## Abstract

Aims: This study aimed to investigate the impact of time-restricted meal intake (TRM) on anthropometric and biochemical parameters in patients with type 2 diabetes mellitus (T2DM).

Methods: A total of 400 patients diagnosed with T2DM were selected from the Endocrinology Department at King George's Medical University (KGMU), Lucknow, based on the American Diabetes Association (ADA) guidelines and specific criteria. A total of 127 patients were lost to follow-up, resulting in 273 patients who completed the study. The patients were randomly assigned to two groups: the TRM group (consenting to have an early dinner at 7 pm) and the control group (non-TRM/late-night eater group). Baseline data were recorded, and follow-up assessments were conducted at six months, 12 months, and 18 months. Informed consent was obtained, and a diet chart was regularly maintained and updated.

Results: The TRM group experienced a significant weight loss of 3.88 kg (5.45%) and a substantial reduction in BMI by 1.5 units (5.26%). In contrast, the non-TRM/control group had smaller reductions in weight (1.36 kg, 1.77%) and BMI (0.5 units, 1.65%). TRM group showed significant reductions in fasting blood sugar levels by 33.9 mg/dl (21.17%), postprandial blood sugar levels by 94.6 mg/dl (38.88%), and glycosylated hemoglobin (HbA1c) levels by 1.37 (15.87%). These improvements were significantly greater than the reductions observed in the control group, which had decreases of 29.3 mg/dl (17.85%) in fasting blood sugar levels, 41.6 mg/dl (16.84%) in postprandial blood sugar levels, and 0.59 (6.89%) in HbA1c levels.

Conclusion: Our findings underscore the potential of TRM as an effective strategy for weight management and glycemic control in patients with T2DM, even in a long-term context. These results support time-restricted eating as a sustainable lifestyle modification for managing chronic metabolic diseases.

## Introduction

Type 2 diabetes mellitus (T2DM) is a widespread health issue affecting millions of individuals worldwide, with a particularly high prevalence in India due to various etiological factors like overweight, obesity, physical inactivity, insulin resistance, genes and family history, genetic mutations, and hormonal diseases. It is estimated that there are approximately 77 million people aged 18 years and above in India who suffer from T2DM. Shockingly, around 25 million individuals are prediabetic, and nearly half of them remain unaware of their diabetic status [1]. Lifestyle interventions, including dietary modifications, play a pivotal role in managing and preventing complications associated with T2DM like nephropathy, retinopathy, neuropathy, and foot ulcers [2]. One emerging dietary approach that has gained attention is time-restricted meal intake (TRM), which involves confining daily food consumption to a defined time window, typically eight to 12 hours, and observing fasting for the remaining hours [3-5].

Dietary patterns profoundly influence the development, progression, and management of T2DM. The present study aims to contribute to the understanding of the effects of TRM on anthropometric and biochemical parameters in individuals with T2DM. Importantly, this study seeks to address a research gap by extending the follow-up period beyond one year, as previous investigations have been limited in this regard. By doing so, we aim to shed light on the long-term potential benefits of TRM in this population.

Theoretical framework

Chronobiology and Metabolic Health

Proper timing of meal intake plays a pivotal role in integrating various physiological processes, including metabolism, energetics, hormonal secretion patterns, physical coordination, and sleep [2]. The circadian system, governed by the suprachiasmatic nucleus (SCN) in the anterior hypothalamus, acts as the master biological clock in mammals and is entrained by light and dark stimuli. The cyclicality of light and dark impacts the SCN through the retinohypothalamic tract, triggered by photo-sensitive retinal ganglion cells expressing the photopigment melanopsin. While the central clock regulates food intake, energy expenditure, and insulin sensitivity, peripheral clocks in tissues such as the gut, adipose tissue, liver, and pancreas control metabolic pathways. Glucose, lipid, and energy metabolism are all under the influence of the circadian system, which up-regulates or down-regulates them at specific times of the day [3,4].

Time-restricted meal intake and circadian alignment

Studies in humans have demonstrated that aligning meal timing with circadian rhythms in metabolism, such as increasing food intake at breakfast and reducing it at dinner, can lead to improved glycemic control, weight loss, lipid profiles, and reduced hunger [5-11]. Meal timing acts as a dominant entraining cue also called zeitgeber for peripheral clocks, subsequently impacting metabolism and the risk factors associated with chronic diseases. Desynchronization between the master clock and peripheral circadian clocks in liver, fat, and skeletal muscle cells may increase the susceptibility to chronic diseases [4].

Molecular Mechanisms in Circadian Regulation

On the molecular level, fasting has been shown to activate the positive limb of the circadian clock, involving circadian locomotor output cycles kaput (CLOCK) and brain and muscle ARNT-like 1 (BMAL1) genes, while feeding activates the negative limb, involving cryptochrome (CRY) and Period (PER) genes. Delaying breakfast by a few hours has been found to delay the expression of PER-controlled genes in human adipose tissue [12]. Conversely, rhythmic feeding has been shown to maintain the circadian rhythms of clock genes in peripheral tissues, even under constant light or darkness conditions or following SCN lesions [13-15]. These findings highlight the powerful entraining cues of the fasting-feeding cycle on peripheral clocks, surpassing the influence of the light-dark cycle [16,17]. Consequently, consuming energy outside the normal feeding phase, such as late-night eating in humans, may disrupt energy balance by resetting peripheral clocks [18].

TRM has emerged as a potential strategy to reprogram the circadian clock, with AMP-activated protein kinase (AMPK) activation during fasting and mammalian target of rapamycin (mTOR) activation during the fed state playing crucial roles. In the fed state, when nutrients are readily available, the mTOR signaling pathway is activated. mTOR, or mechanistic target of rapamycin, plays a crucial role in regulating cellular processes such as growth and metabolism. One of the effects of activated mTOR is the induction of CRY, a protein that is involved in the regulation of circadian rhythms. CRY, in turn, represses the activity of CLOCK:BMAL1, which are the key components of the molecular clock that control the timing of biological processes [19]. On the other hand, during the fasting state, when nutrient levels are depleted, the AMPK pathway is activated. AMPK is a cellular energy sensor that helps maintain energy balance in response to nutrient availability. AMPK activation enhances the phosphorylation of CRY and PER, which are proteins involved in the regulation of the circadian clock. Phosphorylation is a crucial step for the degradation of these proteins, influencing the oscillations of the circadian clock [19].

## Materials and methods

The study included 400 patients diagnosed with T2DM based on the American Diabetes Association's (ADA) guidelines. Patients were selected from the endocrinology outpatient department (OPD) of King George’s Medical University (KGMU), Lucknow, based on specific selection criteria (as shown in Table 1).

**Table 1:** Selection criteria for the enrolment of patients in the study. T2DM: type 2 diabetes mellitus.

	Selection criteria of patients
	Inclusion criteria
1.	Patients aged 25-60 years with a confirmed diagnosis of T2DM attending the endocrinology OPD
2.	Duration of T2DM less than 5 years.
3.	Treatment with oral anti-diabetic medications (metformin, sulfonylurea, gliptin, glitazones) excluding insulin.
4.	Stable anti-diabetic medication regimen for at least one month prior to enrolment.
5.	Willingness to provide written consent and adhere to a time-restricted meal plan.
	Exclusion criteria
1.	Diagnosis of non-T2DM diabetes variants.
2.	Renal impairment (serum creatinine > 1.5 mg/ml) or significant liver disease (aspartate aminotransferase (AST)/alanine transaminase (ALT) >3 times the normal level).
3.	Pregnant or lactating women, and women planning pregnancy.
4.	Participation in another clinical trial within the last 30 days.
5.	History of significant cardiovascular, hepatic, renal, or psychiatric illness, or hemoglobinopathies affecting glycosylated hemoglobin measurement.
6.	Use of sodium-glucose transport protein 2 (SGLT2) inhibitors or glucagon-like peptide-1 receptor agonists (GLP-1 RA).
7.	Regular overnight shift work or excessive alcohol consumption.

Patients were allocated into two groups: the TRM group (case group) and the control/late-night eater/non-TRM group (non-TRM/control group). Group allocation was determined by patients' consent to have an early dinner at 7 pm. The TRM group agreed to the intervention, while the control group did not. Baseline data were recorded, and follow-up assessments were conducted at six months, 12 months, and 18 months. Informed consent was obtained, and a diet chart was regularly maintained and updated. Body weight was measured using a digital scale (Mi Body Composition Scale, Xiaomi, Beijing, China) with a precision of 0.1 kg. Participants were weighed without shoes and in light clothing. Height was measured to the nearest 1 centimeter (cm) using a stadiometer. Body mass index (BMI) was calculated as weight in kilograms divided by height in meters (m) squared. Fasting blood sugar (FBS), postprandial blood sugar (PPBS), and glycosylated hemoglobin (HbA1c) levels were assessed for each patient at baseline, six months, 12 months, and 18 months. Neck, waist, and hip circumference were measured using an inch tape. The patient stood upright and breathed normally during the measurements. A member of the research team positioned the tape measure at the level of the neck for neck circumference, at the navel for waist circumference, and at the broadest part of the hip for hip circumference. The measuring tape was kept parallel to the floor and snugly encircled the patient's torso without causing discomfort. These measurements were recorded for each patient.

The TRM group underwent a single intervention, which involved adjusting the timing of their dinner to around 7 pm in the evening. A member of the research team, experienced in weight management, individually explained TRM intervention to each patient. The session lasted approximately 15 minutes. Patients were instructed to restrict their eating to a 12-hour period each day for the next 18 months. Specifically, they were advised to have dinner at around 7 pm. Detailed guidance was provided to each patient regarding the TRM intervention, including coping with hunger, permissible beverages (only water and medication) during fasting, and study procedures. Patients were asked to maintain a diary to record their daily food intake and bring it during each follow-up visit. They were instructed to record even small dietary items that they had during the course of the day, such as a toffee, a piece of chocolate, or tobacco. The TRM group was allowed to eat ad libitum during the 12-hour eating window, while outside that window, they could only consume water and medications. The time of eating was also logged in the food record. The patients in the TRM group were instructed to have breakfast at 7 am, thus ensuring a 12-hour fast.

Baseline parameters of patients were recorded during the initial visit to the OPD. Follow-up visits were scheduled at six months (first follow-up), 12 months (second follow-up), and 18 months (third follow-up). The patients' progression with TRM was regularly monitored, and compliance was emphasized during each meeting, which happened every 15 days for each patient. The research director reviewed their dietary adherence records, addressed any issues observed, and stressed the importance of adherence.

The study received clearance from the Institutional Ethics Committee, KGMU (reference number: 881/Ethics/R.Cell-18). Prior to participation, written informed consent was obtained from each patient, with approximately 400 subjects aged 25-60 years evaluated, educated, examined, and trained in circadian eating patterns. A proforma was maintained, containing patient details and a diet chart that was updated weekly (from Monday to Monday). All patients, regardless of their group, received standard conventional treatment for T2DM. By following this comprehensive methodology, the study aimed to assess the effects of TRM on patients with diabetes mellitus over an 18-month period.

Enrolment of patients

**Figure 1:**
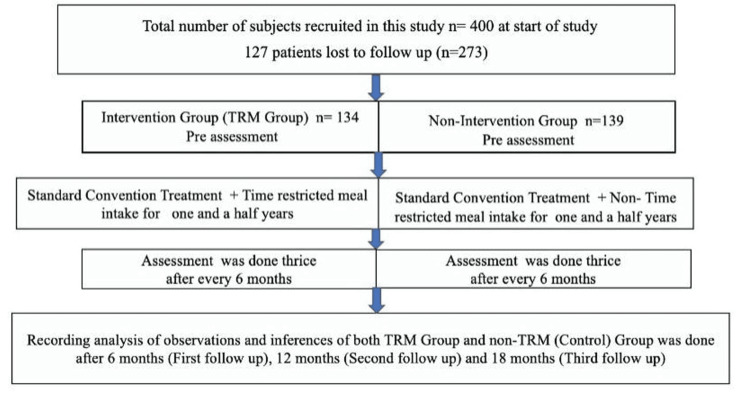
Study design. TRM: time-restricted meal intake. Credit for study design attributed to Dr. Narsingh Verma.

The total duration of the study was from 19 December 2017 to 30 May 2022. The total number of patients recruited in the study from 19 June 2018 to 30 July 2019 was 400, as shown in Figure 1.

Patients lost to follow-up were 127. Total number of patients on whom the study was conducted was 273. A total of 127 patients (31.75% of a total of 400 patients) were lost to follow-up owing to many factors like COVID-19, non-compliance, refusal to pick up calls, change of place, transfers, etc. Out of 134 patients in the TRM group, there were 54 females and 80 males (40.3% females and 59.7% males), and out of 139 patients in the non-TRM/control group, there were 60 females and 79 males (43.16% females and 56.84% males). The parameters measured were anthropometric parameters of age, height (in meters), weight (in kilograms), BMI, neck circumference (in centimeters), waist circumference (in centimeters), and hip circumference (in centimeters), and biochemical parameters of FBS in milligrams per deciliter (mg/dl), PPBS (mg/dl), and HbA1c (%).

Statistics

The normal distribution of the data was assessed, and it was found that our data did not follow a parametric distribution. Therefore, the Mann-Whitney U test was employed to compare the TRM and control groups at baseline and at each stage of follow-up using mean values. We deemed the results to be clinically significant if previously non-significant mean values at baseline became significant at the follow-up assessments or if significant differences between the groups at baseline became highly significant at the subsequent follow-up assessments. Data analysis was conducted using GraphPad Prism 9.2.0 software (GraphPad Software, San Diego, CA). A p-value less than 0.05 (p < 0.05) was considered statistically significant, and a two-tailed p < 0.05 was considered significant.

## Results

The mean age of the patients in the TRM group was found to be 47.17 ± 10.22 years, and the mean age in the non-TRM group was 46.97 ± 10.36 years. In the TRM group, the mean height (in meters) was 1.58 ± 0.08, and in the non-TRM group, the mean height (in meters) was 1.59 ± 0.10.

Weight

**Figure 2:**
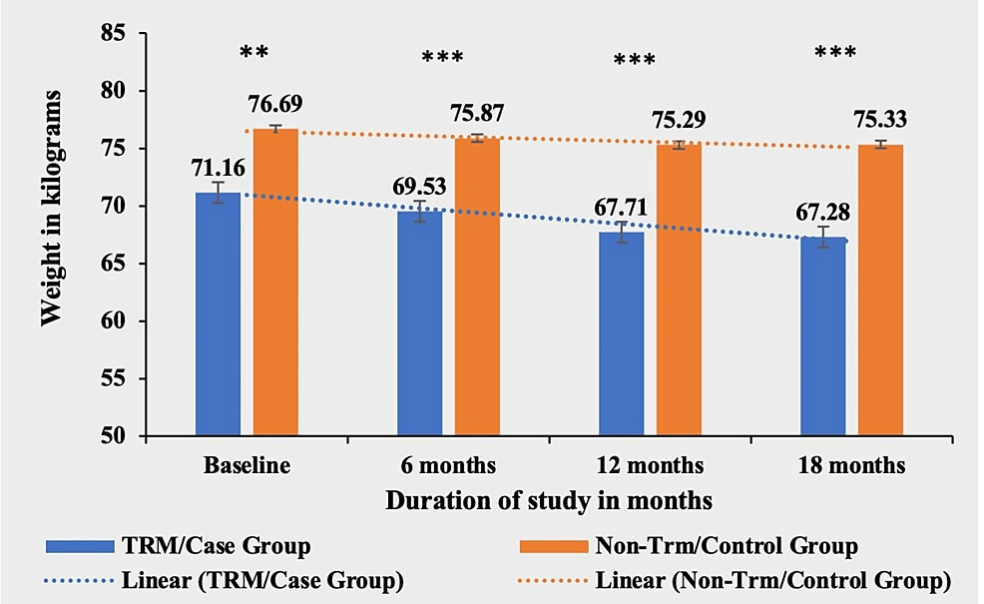
Comparative analysis between TRM/case group and non-TRM/control group on the basis of weight (in kilograms). * p-value < 0.05; ** p-value < 0.001; *** p-value < 0.0001. TRM: time-restricted meal intake.

Patients in the TRM group experienced a significant weight loss of 3.88 kg (5.45%) from their baseline weight of 71.1 ± 12.38 kg. In contrast, the non-TRM group showed a smaller weight loss of 1.36 kg (1.77%). The mean of the weights was significantly different at the baseline between the two groups, but the difference became highly significant in subsequent follow-ups, signifying a greater reduction in weight in the case/TRM group (Table 2 and Figure 2).

**Table 2:** Comparative mean weight in kilograms in both TRM/case group and non-TRM/control group. * p-value < 0.05; ** p-value < 0.001; *** p-value < 0.0001. TRM: time-restricted meal intake.

Weight (in kg)	TRM/case group (N = 134)	Non-TRM/control group (N = 139)	p-value
Baseline	71.16 ± 12.38	76.69 ± 11.33	<0.0003**
6 months	69.53 ± 12.22	75.87 ± 10.83	<0.0001***
12 months	67.71 ± 11.05	75.29 ± 10.66	<0.0001***
18 months	67.28 ± 11.03	75.33 ± 10.55	<0.0001***

Body mass index (BMI)

**Figure 3:**
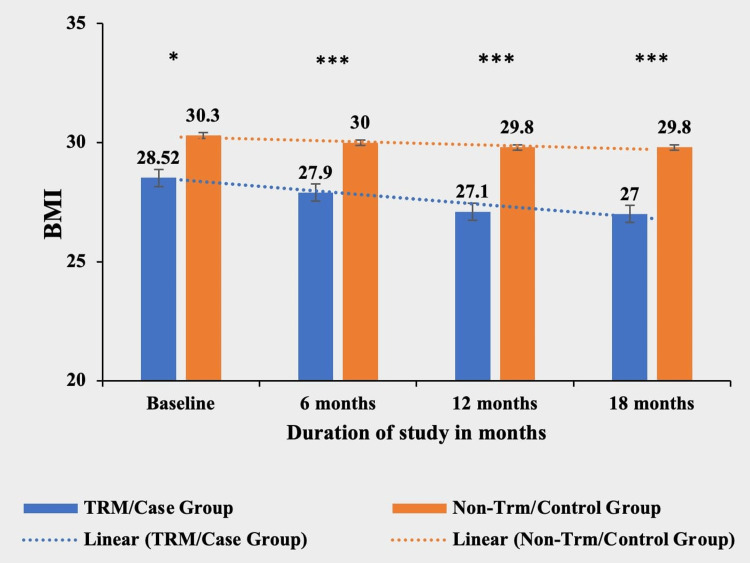
Comparative analysis between TRM/case group and non-TRM/control group on the basis of BMI. * p-value < 0.05; ** p-value < 0.001; *** p-value < 0.0001. TRM: time-restricted meal intake.

Patients in the TRM group demonstrated a statistically significant and clinically meaningful reduction in BMI throughout the study period. The TRM group exhibited a substantial decrease in BMI, with a significant reduction of 1.5 units (5.26%) from their baseline BMI of 28.5 ± 4.97. Conversely, the non-TRM group showed a smaller reduction in BMI of 0.5 units (1.65%) from their baseline BMI of 30.3 ± 3.90. Importantly, the mean BMI values between the TRM and non-TRM groups significantly differed at baseline, indicating initial group disparities. However, as the study progressed, the difference in BMI between the groups became highly significant, demonstrating a greater statistically significant reduction in mean BMI values within the TRM group compared to the non-TRM group (Table 3 and Figure 3).

**Table 3:** Comparative mean BMI in both TRM/case group and non-TRM/control group. * p-value < 0.05; ** p-value < 0.001; *** p-value < 0.0001. TRM: time-restricted meal intake.

BMI	TRM/case group (N = 134)	Non-TRM/control group (N = 139)	p-value
Baseline	28.5 ± 4.97	30.3 ± 3.90	0.0279*
6 months	27.9 ± 7.66	30 ± 8.31	0.0002***
12 months	27.1 ± 4.67	29.8 ± 4.54	0.0001***
18 months	27.0 ± 6.55	29.8 ± 8.71	0.0004***

Neck circumference

**Figure 4:**
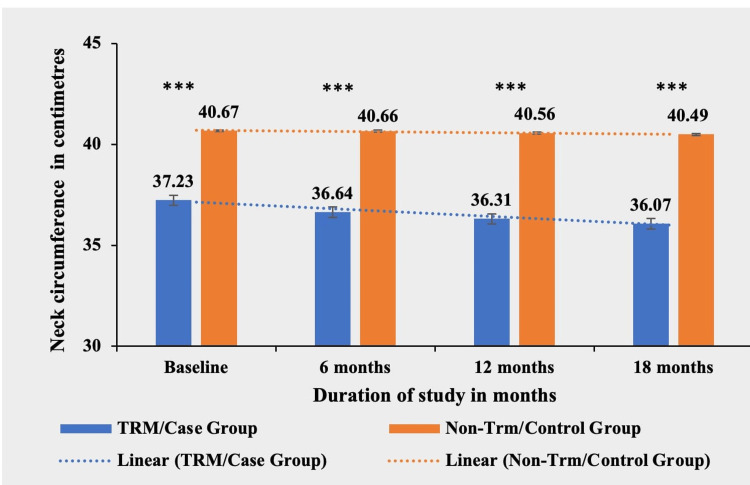
Comparative analysis between TRM/case group and non-TRM/control group on the basis of neck circumference (in centimeters). * p-value < 0.05; ** p-value < 0.001; *** p-value < 0.0001. TRM: time-restricted meal intake.

There was a significant difference in mean neck circumference between the TRM group and the non-TRM group at baseline (p < 0.0001***), and the significance level remained similar in subsequent follow-up assessments, even after reductions in neck circumference. Therefore, the reduction in neck circumference was deemed non-significant between the TRM and non-TRM groups (Table 4 and Figure 4).

**Table 4:** Comparative mean neck circumference (in centimeters) in TRM/case group and non-TRM/control group. * p-value < 0.05; ** p-value < 0.001; *** p-value < 0.0001. TRM: time-restricted meal intake.

Neck circumference (in centimeters)	TRM/case group (N = 134)	Non-TRM/control group (N = 139)	p-value
Baseline	37.23 ± 2.51	40.67 ± 2.93	<0.0001***
6 months	36.64 ± 2.27	40.66 ± 2.93	<0.0001***
12 months	36.31 ± 2.14	40.56 ± 2.91	<0.0001***
18 months	36.07 ± 2.07	40.49 ± 2.90	<0.0001***

Waist circumference

**Figure 5:**
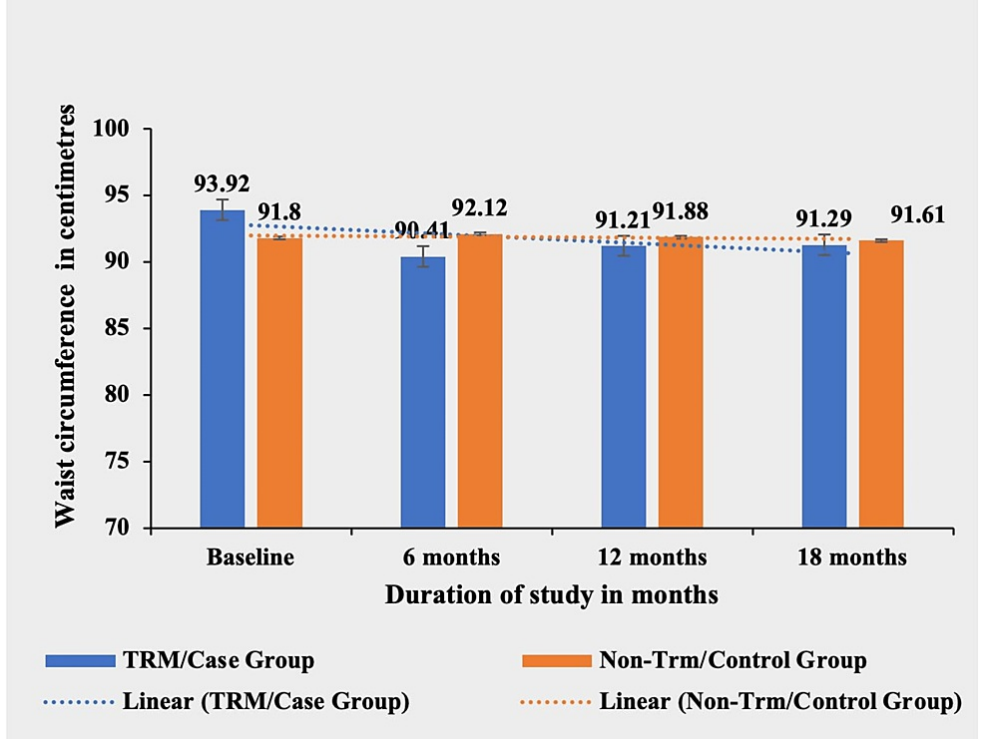
Comparative analysis between TRM/case group and non-TRM/control group on the basis of waist circumference (in centimeters). TRM: time-restricted meal intake.

The waist circumference values for the TRM group and the non-TRM group did not exhibit statistically significant differences throughout the study period (Table 5 and Figure 5).

**Table 5:** Comparative mean waist circumference (in centimeters) in TRM/case group and non-TRM/control group. TRM: time-restricted meal intake; ns: non-significant.

Waist circumference (in centimeters)	TRM/case group (N = 134)	Non-TRM/control group (N = 139)	p-value
Baseline	93.92 ± 14.12	91.80 ± 9.88	0.2466 (ns)
6 months	90.41 ± 10.99	92.12 ± 13.77	0.2821 (ns)
12 months	91.21 ± 8.95	91.88 ± 14.55	0.6504 (ns)
18 months	91.29 ± 14.85	91.61 ± 15.33	0.9596 (ns)

Hip circumference

**Figure 6:**
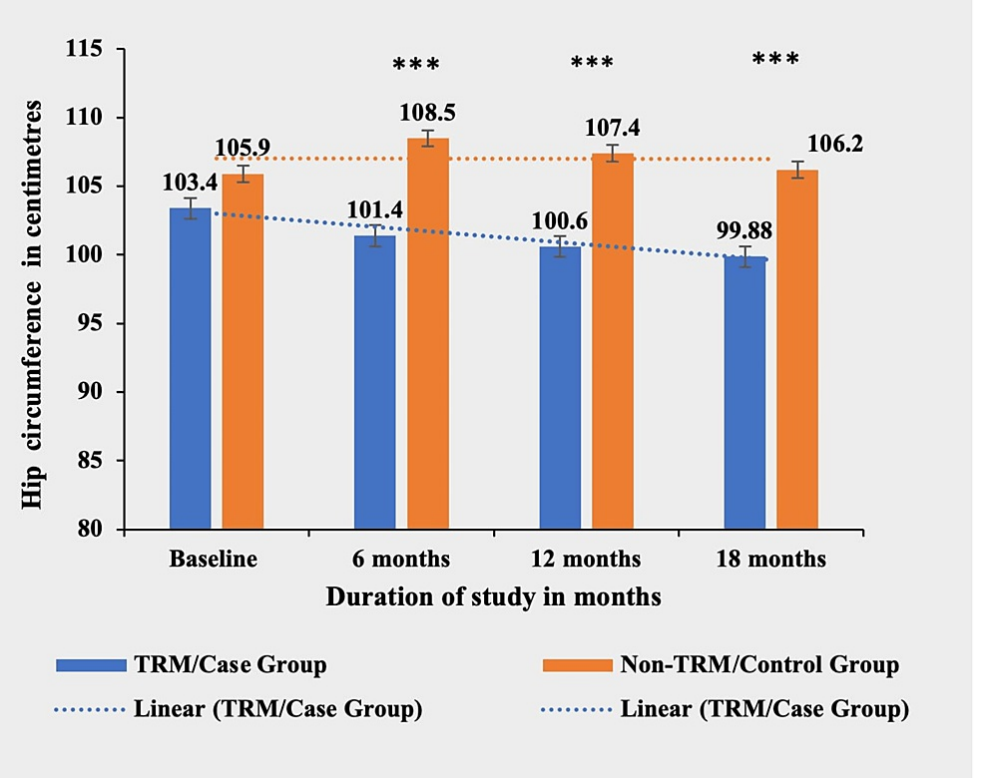
Comparative analysis between TRM/case group and non-TRM/control group on the basis of hip circumference (in centimeters). * p-value < 0.05; ** p-value < 0.001; *** p-value < 0.0001. TRM: time-restricted meal intake.

The TRM group exhibited a significant reduction in hip circumference compared to the non-TRM group at six, 12, and 18 months (p < 0.0001***). The TRM group achieved a mean decrease of 3.6 cm (3.48%) from baseline, while the non-TRM group had a mean increase of 0.3 cm (0.28%) during the study period. There was no significant difference in hip circumference between the two groups at baseline (p = 0.1030) (Table 6 and Figure 6).

**Table 6:** Comparative mean hip circumference (in centimeters) in both TRM/case group and non-TRM/control group. * p-value < 0.05; ** p-value < 0.001; *** p-value < 0.0001. TRM: time-restricted meal intake; ns: non-significant.

Hip circumference (in centimeters)	TRM/case group (N = 134)	Non-TRM/control group (N = 139)	p-value
Baseline	103.4 ± 11.00	105.9 ± 11.77	0.1030 (ns)
6 months	101.4 ± 9.31	108.5 ± 14.3	<0.0001***
12 months	100.6 ± 6.54	107.4 ± 9.7	<0.0001***
18 months	99.8 ± 8.27	106.2 ± 9.58	<0.0001***

Blood sugar fasting

**Figure 7:**
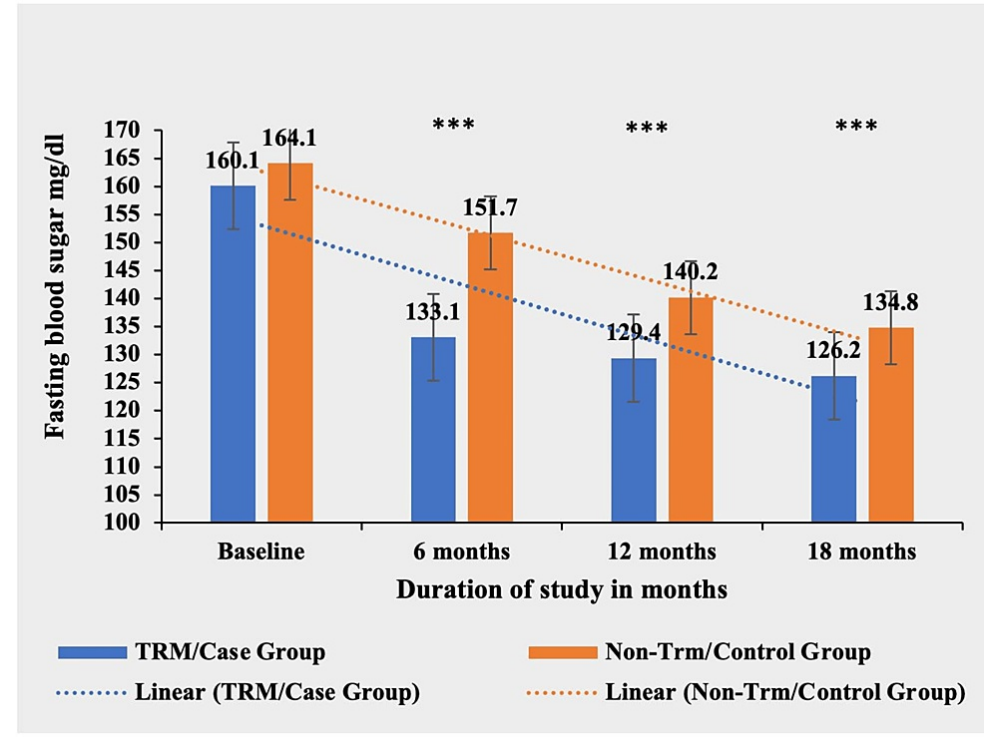
Comparative analysis between TRM/case group and non-TRM/control group on the basis of fasting blood sugar. * p-value < 0.05; ** p-value < 0.001; *** p-value < 0.0001. TRM: time-restricted meal intake.

The TRM group demonstrated a significant reduction in FBS levels compared to the non-TRM group at six, 12, and 18 months (p < 0.0001***). The TRM group achieved a mean decrease of 33.9 mg/dl (21.17%) from baseline, while the non-TRM group had a mean decrease of 29.3 mg/dl (17.85%) during the study period. There was no significant difference in FBS levels between the two groups at baseline (p = 0.0505) (Table 7 and Figure 7).

**Table 7:** Comparative mean blood sugar fasting (mg/dl) in TRM/case group and non-TRM/control group. * p-value < 0.05; ** p-value < 0.001; *** p-value < 0.0001. TRM: time-restricted meal intake; ns: non-significant.

Blood sugar fasting (mg/dl)	TRM/case group (N = 134)	Non-TRM/control group (N = 139)	p-value
Baseline	160.1 ± 20.68	164.1 ± 21.20	0.0505 (ns)
6 months	133.1 ± 17.41	151.7 ± 19.42	<0.0001***
12 months	129.4 ± 13.89	140.2 ± 18.16	<0.0001***
18 months	126.2 ± 14.54	134.8 ± 17.19	<0.0001***

Postprandial blood sugar

**Figure 8:**
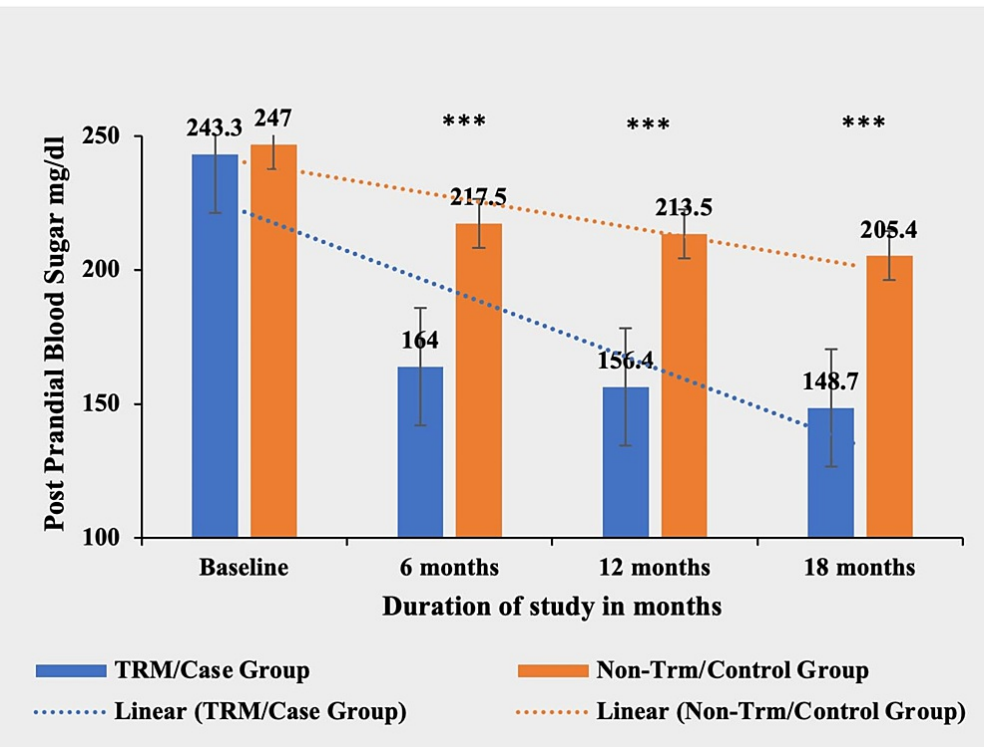
Comparative analysis between TRM/case group and non-TRM/control group on the basis of postprandial blood sugar. * p-value < 0.05; ** p-value < 0.001; *** p-value < 0.0001. TRM: time-restricted meal intake.

The TRM group exhibited a significant reduction in PPBS levels compared to the non-TRM group at six, 12, and 18 months (p < 0.0001***). The TRM group achieved a mean decrease of 94.6 mg/dl (38.88%) from baseline, while the non-TRM group had a mean decrease of 41.6 mg/dl (16.84%) during the study period. There was no significant difference in PPBS levels between the two groups at baseline (p = 0.6777) (Table 8 and Figure 8).

**Table 8:** Comparative mean postprandial blood sugar in TRM/case group and non-TRM/control group. * p-value < 0.05; ** p-value < 0.001; *** p-value < 0.0001. TRM: time-restricted meal intake; ns: non-significant.

Postprandial blood sugar (mg/dl)	TRM/case group (N = 134)	Non-TRM/control group (N = 139)	p-value
Baseline	243.3 ± 64.83	247 ± 38.22	0.6777 (ns)
6 months	164 ± 40.34	217.5 ± 37.07	<0.0001***
12 months	156.4 ± 35.9	213.5 ± 36.6	<0.0001***
18 months	148.7 ± 35.86	205.4 ± 37.32	<0.0001***

Glycosylated hemoglobin (HbA1c)

**Figure 9:**
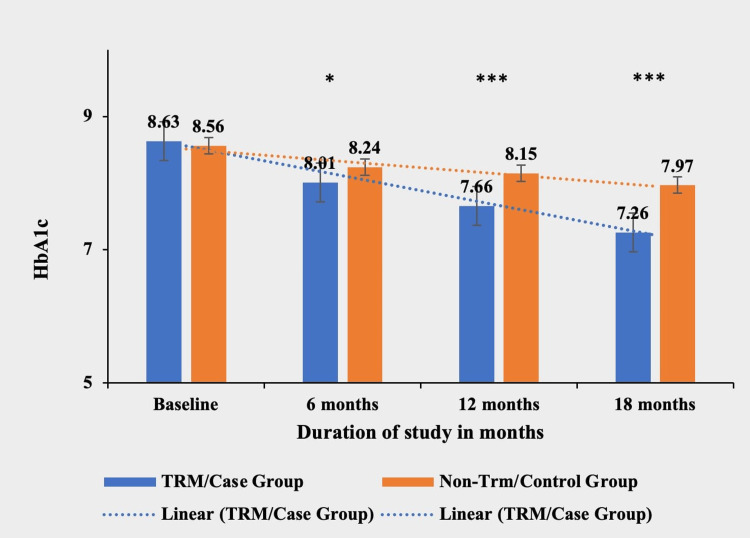
Comparative analysis between TRM/case group and non-TRM/control group on the basis of HbA1c. * p-value < 0.05; ** p-value < 0.001; *** p-value < 0.0001. TRM: time-restricted meal intake; HbA1c: glycosylated hemoglobin.

The TRM group demonstrated a significant reduction in HbA1c levels compared to the non-TRM group at six, 12, and 18 months (p < 0.0001***). The TRM group achieved a mean decrease of 1.37 (15.87%) from baseline, while the non-TRM group had a mean decrease of 0.59 (6.89%) during the study period. There was no significant difference in HbA1c levels between the two groups at baseline (p = 0.4584) (Table 9 and Figure 9).

**Table 9:** Comparative mean HbA1c in both TRM/case group and non-TRM/control group. * p-value < 0.05; ** p-value < 0.001; *** p-value < 0.0001. TRM: time-restricted meal intake; HbA1c: glycosylated hemoglobin; ns: non-significant.

HbA1c	TRM/case group (N = 134)	Non-TRM/control group (N = 139)	p-value
Baseline	8.63 ± 10.64	8.56 ± 7.82	0.4584 (ns)
6 months	8.01 ± 8.66	8.24 ± 9.83	0.0159*
12 months	7.66 ± 6.67	8.15 ± 6.80	<0.0001***
18 months	7.26 ± 7.59	7.97 ± 5.78	<0.0001***

## Discussion

Patients in the TRM/case group exhibited significant improvements in various health parameters compared to the non-TRM/control group. Such synchronization could potentially improve glycemic control and weight management, two critical aspects in the management of diabetes. The TRM group experienced a significant weight loss of 3.88 kg (5.45%) and a substantial reduction in BMI by 1.5 units (5.26%). In contrast, the non-TRM/control group had smaller reductions in weight (1.36 kg, 1.77%) and BMI (0.5 units, 1.65%). Additionally, the TRM group showed significant reductions in FBS levels by 33.9 mg/dl (21.17%), PPBS levels by 94.6 mg/dl (38.88%), and HbA1c levels by 1.37 (15.87%). These improvements were significantly greater than the reductions observed in the control group, which had decreases of 29.3 mg/dl (17.85%) in FBS levels, 41.6 mg/dl (16.84%) in PPBS levels, and 0.59 (6.89%) in HbA1c levels. These findings suggest that implementing TRM may improve weight management and better glycemic control in individuals with T2DM.

This study aimed to evaluate the impact of TRM on key health parameters in individuals with T2DM. Our results suggest that TRM has significant implications for managing this condition, which aligns with previous research. A statistically significant reduction in weight and BMI was observed among patients in the TRM group compared to the non-TRM/control group. These findings are supported by past studies, which also showed weight loss and BMI reduction due to restricted meal intake within a specific time window [20,21].

Interestingly, the degree of weight loss accomplished in our study (5.45% of baseline weight) is higher than the typical 1-3% reductions seen in other time-restricted eating clinical trials [20]. This is further supported by Kahleova et al.'s comprehensive study, which reported a lower BMI associated with fewer daily meals and more extended overnight fasting [21].

Insulin sensitivity, a key factor in blood sugar control, exhibits diurnal variations, decreasing throughout the day and into the night. This diurnal pattern is partly due to the circadian rhythm of insulin secretion and the inhibitory effect of growth hormone, which tends to increase during nighttime [22]. As a result, postprandial insulin and glucose responses to meals tend to increase later in the day and into the night [23-27]. Consuming meals at night is associated with greater postprandial glucose and insulin exposure compared to meals consumed during the day [28]. Studies have shown that inducing circadian misalignment, such as extending the day from a 24-hour to a 28-hour cycle, can lead to insulin resistance after only a few cycles [4]. Feeding regimens may improve the oscillations in circadian clock gene expression, reprogram molecular mechanisms of energy metabolism, and contribute to improved regulation of body weight. During the overnight fast, a metabolic switch occurs in fuel utilization, shifting from glucose to ketones [29,30]. Ketones are produced by the liver through the process of ketogenesis [31], utilizing fatty acids as a substrate; this reinforces the circadian rhythms of metabolism and has been associated with reduced oxidative stress and inflammation [32]. In TRM, a fasting period of 12 to 16 hours per 24-hour cycle is achieved, which stimulates pathways linked to long-term fasting approaches, such as autophagy [33]. TRM has been shown to improve metabolism regardless of dietary macronutrient composition or energy restriction [34].

TRM has emerged as a promising dietary intervention for managing T2DM, demonstrating benefits in glycemic control, insulin sensitivity, and overall cardio-metabolic health. Studies have shown that TRM can lead to significant improvements in glycemic control, as evidenced by reductions in fasting plasma glucose (FPG), HbA1c, and other cardiometabolic risk factors in patients with impaired fasting glucose, a precursor to T2DM [35]. Further, the impact of TRM on insulin sensitivity and metabolism in T2DM patients has been explored, revealing that extending the overnight fast can improve fasting and 24-hour glucose levels without notably affecting fat oxidation or hepatic glycogen stores [36]. Comparatively, TRM has been shown to be as effective as a low-carbohydrate diet in managing mean glucose levels in T2DM patients [37]. Significant weight reduction and modest weight loss have been reported in obese individuals and those at risk of T2DM following a TRM schedule, indicating its effectiveness in weight management [38,39]. Studies have also highlighted reductions in both fasting and postprandial glucose levels and a decrease in HbA1c levels, suggesting improved long-term glycemic control in pre-obese and obese diabetic patients following early 10-hour TRM [40]. While some studies have noted contradictory results, the overall evidence suggests the beneficial effects of TRM on glucose and lipid metabolism [41]. An umbrella review further reinforces the potential of TRM in complementing usual care for weight and glycemic control [42]. Lastly, TRM's ability to improve insulin sensitivity and glucose tolerance in subjects at risk of T2DM suggests its utility in preventing the disease [43].

Previous research has underscored the impact of meal timing on metabolic processes, indicating that synchronizing meal intake with circadian rhythms might be beneficial for patients with T2DM [3-5]. The available literature adequately establishes that TRM improves metabolic outcomes in humans [19], with most studies reporting modest reductions in body weight and fat mass [6,44-50].

A noteworthy aspect of our results is the significant decrease in FBS and PPBS, as well as HbA1c levels in the TRM group. Several prior studies have also highlighted the potential role of meal timing in regulating blood glucose levels [51,52]. In a study conducted by Jamshed et al. [52], early time-restricted feeding (eTRF) was found to alter the temporal patterns of 24-hour glucose levels as measured by continuous glucose monitoring (CGM). Eleven overweight adults participated in a four-day randomized crossover study where they ate between 8 am and 2 pm (eTRF) and between 8 am and 8 pm (control schedule). When averaged across the entire day, eTRF led to a reduction in mean 24-hour glucose levels by 4 ± 1 mg/dl (p = 0.0003) [52]. Sutton et al. [53] showed positive effects for insulin markers [50], whereas the study by Hutchison et al. [51] showed positive effects for glucose markers. Additionally, the study by Jamshed et al. showed clear effects through glucose markers, but the results regarding insulin markers were conflicting [52]. Overall, the current findings support the conclusion that all studies demonstrated a beneficial effect of TRM on the glycemic profile by at least one glycemic index (glucose or insulin). Therefore, this new dietary pattern could be a promising strategy for preventing and/or treating chronic metabolic diseases such as T2DM [52].

Explaining these results involves considering the circadian rhythm and its influence on metabolic processes. TRM is thought to harmonize with the body's natural circadian rhythm, leading to improved metabolic outcomes and, thereby, better control of weight and glycemic parameters. By narrowing the window of food intake, it might also reduce the duration of insulin production, potentially enhancing insulin sensitivity and glycemic control [52].

Despite these positive findings, our study also revealed no significant change in waist circumference among both the TRM and non-TRM/control groups, contrasting with some previous investigations demonstrating a significant reduction with time-restricted feeding [54]. The substantial decrease in hip circumference seen in the TRM group suggests a loss of subcutaneous fat, which has previously been associated with time-restricted eating [54]. The slight variations among these findings might stem from variations in the intervention's duration, intensity, patient adherence, or the influence of other confounding factors.

One of the significant contributions of our study lies in its exploration of the long-term effects of TRM, an area often overlooked in previous research. Indeed, while several studies have established the short-term benefits of TRM [20,51-57], there is a dearth of information about its enduring effects, especially when implemented over a period exceeding one year. Importantly, our findings demonstrated that the influence of TRM on key health parameters is not just persistent but also incremental in nature. Weight loss [58,59], reduction in BMI, and improvements in glycemic control all showed sustained decreases throughout the study duration, suggesting that the benefits of TRM potentially compound over time. This aspect of our study is of particular importance for individuals with chronic metabolic conditions, such as T2DM, where management is typically long term, and lifestyle modifications must be sustainable for patients to experience enduring benefits. Judicious use of this fasting and feeding regimen is dependent on the in-depth guidance, motivation, and counseling provided by the medical practitioner and the dietitian or nutritionist present in the clinical setting. On the basis of our team's experience, while guiding and counseling the patient, we observed that once TRM becomes a habit, then the patient usually can and will follow it regularly but the first month is crucial wherein the patient might need additional counseling and encouragement to keep following this healthy habit of TRM.

Limitations

During the course of this study, we acknowledge certain limitations that may have affected the reliability of our findings. One notable limitation is the reliance on self-reported dietary intake by the patients. Due to resource constraints and the large number of patients involved, it was not feasible to monitor all 273 patients under strict supervision for the entire 18-month duration of the study. Instead, we maintained regular communication with the patients through telephone calls, emails, and messages at intervals of seven to eight days. However, adherence to the time-restricted feeding regimen primarily relied on the willingness and commitment of the patients, which introduces a potential source of error and variability in the data.

The self-reporting of dietary intake is subject to recall bias and inaccuracies, as individuals may unintentionally misreport their food consumption. Moreover, the level of detail and accuracy in reporting may vary among patients. These limitations in dietary assessment could have influenced the interpretation of the results and the overall efficacy of the time-restricted feeding intervention.

To mitigate these limitations in future studies, more rigorous monitoring methods such as direct observation or the use of wearable devices for dietary tracking could be implemented. Additionally, strategies to enhance adherence and compliance to the prescribed regimen should be explored, such as providing regular counseling, support, and incentives to patients.

Despite these limitations, our study still provides valuable insights into the potential effects of time-restricted feeding. However, further research with improved methodologies and larger sample sizes is necessary to validate our findings and address the existing gaps in knowledge. However, long-term adherence to TRM may present social challenges. Future research should explore shorter fasting periods, include "days off" to improve sustainability, and also consider the individual chronotypes while maintaining the intervention's benefits.

## Conclusions

In conclusion, our study contributes valuable insights into the potential benefits of TRM in the management of T2DM, specifically among Indian patients with T2DM over an 18-month follow-up period. The findings underscore TRM as a promising and sustainable solution for addressing issues related to obesity and blood sugar control. The demonstrated weight loss of 3.88 kg (5.45%) among TRM adherents, coupled with statistically significant improvements in BMI, hip circumference, and key blood sugar markers, highlights the efficacy of this feeding regimen. The notable improvements in FBS and PPBS levels, as well as HbA1c values, indicate the potential of TRM as an adjunctive approach alongside prescribed medications for comprehensive diabetes care.

It is noteworthy that no adverse effects were observed in our study participants, which might be due to strict adherence to the selection criteria; however, the successful adoption of TRM requires careful guidance, motivation, and counseling by medical practitioners, dieticians, or nutritionists, particularly during the initial month when patients may need additional support to establish this healthy habit. In light of these findings, time-restricted feeding emerges as a safe, convenient, and straightforward dietary strategy that holds promise not only for individuals managing T2DM but also for those seeking effective weight loss methods. As we look toward the future, further research involving diverse patient populations, individualizing TRM protocols based on patients' chronotypes, and exploration of additional health parameters will be crucial for validating and expanding upon the positive outcomes observed in our study.
